# Photocatalytic and Antimicrobial Properties of Electrospun TiO_2_–SiO_2_–Al_2_O_3_–ZrO_2_–CaO–CeO_2_ Ceramic Membranes

**DOI:** 10.1021/acsomega.2c06986

**Published:** 2023-03-17

**Authors:** Nuray Yerli Soylu, Anıl Soylu, Dilara Nur Dikmetas, Funda Karbancioglu-Guler, Sadriye Kucukbayrak, Melek Erol Taygun

**Affiliations:** † Faculty of Chemical and Metallurgical Engineering, Department of Chemical Engineering, Istanbul Technical University, Maslak, Istanbul 34449, Turkey; ‡ Faculty of Mines, Department of Mining Engineering, Istanbul Technical University, Maslak, Istanbul 34449, Turkey; § Faculty of Chemical and Metallurgical Engineering, Department of Food Engineering, Istanbul Technical University, Maslak, Istanbul 34449, Turkey; ⊥ Faculty of Engineering, Department of Chemical Engineering, Marmara University, Maltepe, Istanbul 34854, Turkey

## Abstract

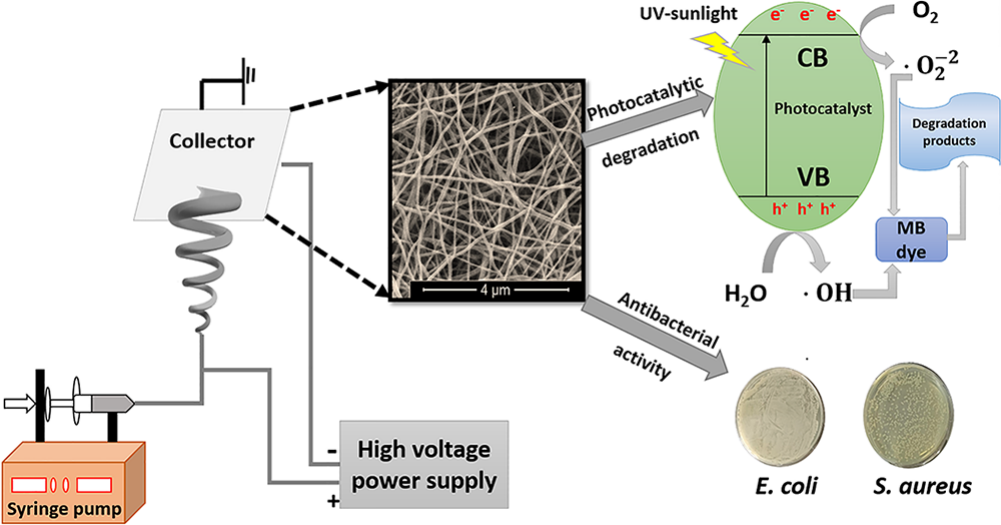

In this study, TiO_2_-based ceramic nanofiber membranes in the system of TiO_2_–SiO_2_–Al_2_O_3_–ZrO_2_–CaO–CeO_2_ were synthesized by combining sol–gel and electrospinning processes. In order to investigate the thermal treatment temperature effect, the obtained nanofiber membranes were calcined at different temperatures ranging from 550 to 850 °C. Different characterization methods such as X-ray diffraction (XRD), scanning electron microscopy (SEM), Fourier transform infrared (FT-IR), and high-resolution transmission electron microscopy (HR-TEM) were conducted on the obtained membranes to investigate the structural and morphological properties of the nanofibers. The Brunauer–Emmett–Teller surface area of the nanofiber membranes was very high (46.6–149.2 m^2^/g) and decreased with increasing calcination temperature as expected. Photocatalytic activity investigations were determined using methylene blue (MB) as a model dye under UV and sunlight irradiation. High degradation performances were achieved with the calcination temperatures of 650 and 750 °C because of the high specific surface area and the anatase structure of the nanofiber membranes. Moreover, the ceramic membranes showed antibacterial activity against *Escherichia coli* as a Gram-negative bacterium and *Staphylococcus aureus* as a Gram-positive bacterium. The superior properties of the novel TiO_2_-based multi-oxide nanofiber membranes proved as a promising candidate for various industries, especially the removal of textile dyes from wastewater.

## 1 Introduction

The growth of industrialization and the increase in population bring out environmental pollution.^1,2^ Organic pollutant emissions, especially from textile dyes in wastewater, pose a serious threat to certain organisms and human health, leading to toxic effects due to their poisonous nature and nonbiodegradable properties. Removal of these organic dyes from industrial wastewater has become a serious problem, and the selection of the best treatment method plays a vital role in the environment and humanity.^3^ Various treatment methods, such as physical methods, biological treatment, chemical oxidation, and electrochemical oxidation, are proposed to overcome the disposal problem of these organic pollutants.^4,5^ However, organic dyes have a biopersistent nature and are strongly resistant to most treatment techniques, and some of these methods cause secondary pollution.^2,6,7^ In recent years, semiconductor-based photocatalysis has emerged as a promising green technology for the degradation of organic pollutants from textile wastewater due to its unique properties such as being environmentally friendly and cost effective.^2,3,8^ Among different semiconductor materials for photocatalytic processes in water treatment applications, TiO_2_, especially in the anatase phase, is the most preferred photocatalyst due to its superior properties such as being inexpensive, nontoxic for the human body and environment, photochemically stable, and relatively easy to fabricate.^4,9−12^ TiO_2_-based materials are frequently preferred in many technical and electronic applications apart from this field.^13^ However, the relatively large band gap energy (3.2 eV) limits its use to only ultraviolet (UV) irradiation, representing 5% of solar energy.^14^

In order to provide an efficient dye degradation process, decreasing the electron–hole recombination rate and enhancing solar energy utilization are critical issues. Recently, it has been proposed to combine TiO_2_ with different metals, nonmetals, and lanthanide ions to eliminate disadvantageous properties. Suitable dopant selection plays a vital role in reaching a high degradation rate of dyes under sunlight irradiation.^12,14^ Among the different rare-earth metal ions for doping elements, CeO_2_ is the most preferred dopant owing to its properties such as being nontoxic, low cost, capability of strong oxidation, nonphotocorrosive, high conductivity, and high chemical stability.^7,15−18^ Many studies have been used to improve degradation properties of the photocatalysts for different dyestuffs under UV and solar light by the help of CeO_2_-doped TiO_2_ photocatalysts.^16−24^ Apart from rare earth elements, the photocatalytic degradation efficiency of TiO_2_ could be enhanced by doping an another n-type semiconductor as ZrO_2_. The ionic radius and electronegativity of zirconium (Zr^4+^) and titanium (Ti^4+^) are similar. The addition of zirconia induces oxygen defects and the diversity of Ti^4+^ to Zr^4+^ which leads to the improvement of photocatalytic activity by extending the absorption range from UV to visible light.^14,25^ Although zirconia doping brings many good features, single doping has some limitations as restricted improvement for the visible light response of photocatalysts.^26^ Therefore, researchers have been concentrated on co-doping instead of single doping of zirconia to the TiO_2_ photocatalyst to reach the maximum benefit for the photocatalytic degradation process under UV and sunlight irradiation.^14,25−28^ On the other hand, stabilizing the anatase phase of TiO_2_ materials is another problem in photocatalytic processes. The doping of Ca^2+^ to the TiO_2_ ensures the reduction of the rutile, and thus, better photocatalytic activity could be provided. Minchi et al. studied Ca-doped TiO_2_ nanomaterials, and they reached higher photocatalytic activity than pure TiO_2_, thanks to the stabilization of the anatase phase by Ca doping.^29^ Also, SiO_2_ plays a vital role in the production of ceramic materials. SiO_2_-doped TiO_2_ nanomaterials have excellent thermal properties and chemical durability. Doping of SiO_2_ to the nanofiber structure causes delay in the anatase to rutile phase transformation for TiO_2_, even at high calcination temperatures.^30−32^

In photocatalytic applications, the efficiency of doped TiO_2_ materials depends on their shape and surface area. The use of porous nanostructured materials such as nanofibers, nanotubes, nanoparticles, and nanosheets with different properties provides advantages in many different application areas such as gas sensors, supercapacitors, and water treatment applications.^12,33,34^ Nanostructured materials, especially TiO_2_ in the nanoparticle form or thin films, were used to achieve the best water treatment performance.^35^ Although TiO_2_ nanoparticles have a large surface area, their fixation and recovery are hardly difficult, and it causes secondary pollution in water. In order to overcome this problem, TiO_2_ nanomaterials were prepared by coating on different substrates such as glass plates or ceramic materials. However, it leads peeling problem of coated TiO_2_ from the substrate surface. To be able to solve this problem, TiO_2_ can be incorporated directly into the glass–ceramic system. Glass ceramics attract much attention due to their low-cost production and can be prepared easily in various sizes and shapes, such as nanorods and nanofibers.^31,36^ Electrospinning is a simple, inexpensive, and relatively versatile technique to fabricate glass ceramic nanomaterials which have unique properties such as a high surface area to volume ratio, flexibility, highly porous structure, and easy use, and it is possible to control the nanofiber composition to reach desired results from its properties.^37−39^ In recent years, novel doped TiO_2_ glass–ceramic nanofiber membranes have been prepared with the aid of sol–gel and electrospinning techniques for the photocatalytic degradation of organic pollutants.^32,40,41^ The nanofibrous structure of doped TiO_2_ materials not only improves adsorption capacity but also prevents secondary pollution due to its morphology.^42^ In this context, many studies have been realized by doping TiO_2_ with different materials by combining sol–gel and electrospinning processes. Tobin et al. synthesized TiO_2_–Al_2_O_3_ fibers, and doping alumina to TiO_2_ structure provided the stability of the anatase phase at even high temperatures.^43^ Lotus et al. reported that the electrospun TiO_2_–Al_2_O_3_ nanofibers, which calcined at high temperatures, preserved the anatase phase of TiO_2_, and they claimed that the improvement of photocatalytic efficiency could be reached by alumina doping.^40^ Also, Ismail et al. reported that TiO_2_–Al_2_O_3_ nanocomposites showed better photocatalytic efficiency than pristine titania.^44^ Frontera et al. produced nickel, niobium, and tantalum-doped TiO_2_ with a high surface area by the sol–gel and electrospinning process to investigate the crystalline structure.^45^ Moreover, nanocomposites such as TiO_2_–Er,^46,47^ TiO_2_–SiO_2_,^15,32,41^ Eu^3+^-doped TiO_2_–SiO_2_,^30^ TiO_2_–CeO_2_,^23^ Li–TiO_2_,^48^ TiO_2_–Ca,^29,48^ Fe_2_O_3_–TiO_2_,^49^ and TiO_2_/g-C_3_N^50^ were produced from sol–gel and polymer mixture using the electrospinning method and calcination processes.

Antimicrobial activity is another important factor in producing efficient nanomaterials for biosafety and sustainable applications. Due to their high qualitative properties such as flexibility, high surface area to volume ratio, and high porosity with continuously interconnected pores, electrospun nanofibers can be good candidates for disinfection applications.^51^ TiO_2_ nanomaterials, especially in the anatase phase, display antibacterial activity but they have limited inhibition to the growth of antibiotic-resistant bacteria.^52,53^ Doping of TiO_2_ enhances not only photocatalytic activity but also improves the antibacterial properties of TiO_2_ nanomaterials, even in the absence of a light source. Moongraksathum et al. synthesized TiO_2_ co-doped with silver and ceria, and they found high antimicrobial activity against *E. coli* and *S. aureus* both under UV light and dark conditions.^54^ Hassan et al. reached high bactericidal efficiency with electrospun Ce_2_O_3_–TiO_2_ nanofibers on Gram-positive and Gram-negative microorganisms, and they commented that the killing of bacteria was originated by cerium oxide atoms.^55^

In the present study, it was aimed to improve the antibacterial properties of nanomaterials, including TiO_2_ with the help of the synergistic effect of doping materials. To the best of our knowledge, TiO_2_ has been doped one or two oxides to overcome its drawbacks for the efficient photocatalytic processes. However, in the present study, it was aimed to synthesize the TiO_2_-based multi-oxide ceramic nanofiber membrane by direct incorporation of the doping materials into the sol–gel solution and using the electrospinning method. It was purposed to benefit the synergistic effect of different oxides, not only utilize the enhancement of photocatalytic activity under both UV and sunlight irradiation but also ensure the antibacterial properties of TiO_2_ nanofibers.

## 2 Experimental Section

### 2.1 Materials

Titanium(IV) isopropoxide (TTIP ≥ 97%, Aldrich), tetraethyl orthosilicate (TEOS, 98%, Aldrich), aluminum nitrate nonahydrate (Al(NO_3_)_3_·9H_2_O, 98%, Merck), calcium nitrate tetrahydrate (Ca(NO_3_)_2_·4H_2_O, Merck), cerium(III) nitrate hexahydrate (Ce(NO_3_)_3_·6H_2_O, (99.999% trace metals basis, Aldrich), and zirconyl chloride octahydrate **(**ZrOCl_2_·8H_2_O, 98%, Merck) were used without any purification as TiO_2_, SiO_2_, Al_2_O_3_, CaO, CeO_2_, and ZrO_2_ sources. Dimethylformamide (DMF > 98%, Merck) was chosen as the solvent instead of water because of the fact that polyacrylonitrile (PAN *M*_w_ = 150,000 Da, Sigma) is a hydrophobic polymer. Triton-X (98–100%, Merck) solution was used as a surfactant. Methylene blue (C_16_H_18_ClN_3_S·*x*H_2_O, Merck) was used as a model dye pollutant.

### 2.2 Preparation of Sol–Gel Solution and Fabrication of Nanofibers by the Electrospinning Method

The glass–ceramic nanofiber membrane in the system of 65%TiO_2_–20%SiO_2_–5%Al_2_O_3_–5%ZrO_2_–3%CaO-2%CeO_2_ (in wt %) was fabricated by combined sol–gel and electrospinning techniques. First, Al(NO_3_)_3_·9H_2_O was added into DMF under continuously stirring conditions. After completely dissolving, Ca(NO_3_)_2_·4H_2_O, ZrOCl_2_·8H_2_O, and Ce(NO_3_)_3_·6H_2_O were added, respectively, and it was provided entirely to dissolve. Then, TEOS was added into solution drop by drop. After adding 1 M HCl, the solution was stirred for 45 min. As the last ingredient, TTIP was added drop by drop, and the solution was stirred for 24 h. The prepared sol was yellow and transparent. On the other hand, 10 wt % PAN solution was prepared by using DMF as a solute under continuously stirring for 24 h. As the last step, the sol and polymer solutions were mixed with each other at a ratio of 1/4 (w/w). The obtained solution was stirred for 24 h before the electrospinning process.

As-prepared solutions were loaded into a plastic syringe equipped with a flat stainless-steel needle. This needle was connected to a high-voltage supply. The electrospinning process was realized by an electrospinning device (Nanospinner 24 Touch, Inovenso Co.) under specific process conditions. The applied voltage was set at 20 kV, and the feeding flow rate was adjusted as 2 mL/h by taking the needle tip to the collector distance as 130 mm. The electrospun nanofibers were deposited as nonwoven mats on a grounded target wrapped with aluminum foil. The electrospinning process was performed at ambient temperature with a relative humidity of 45–55%. The obtained nanofibers were dried in a vacuum oven at 60 °C for 24 h. Finally, the dried samples were calcined at different temperatures between 550–850 °C for 1 h in a muffle furnace with a heating rate of 2 °C/min to remove nitrate residues and to form a ceramic structure.

### 2.3 Characterization

Different characterization methods were applied to investigate the prepared nanofiber samples. The crystal structures of the nanofiber samples were investigated by using a Panalytical Xpert Pro X-ray diffractometer with Cu-Kα radiation (wavelength = 0.15419 nm). XRD data of each nanofiber were accumulated over the 2θ range from 5 to 80° with a step size of 0.017°. Fourier transform infrared (FT-IR) spectroscopy was used to investigate the structural composition of the obtained nanofibers. Spectra were collected using a Perkin Elmer Spectrum 100 Model spectrometer in transmittance mode between 4000 and 650 cm^–1^. Scanning electron microscopy (SEM, JSM-5410, Jeol) was used to determine the surface morphologies and the microstructure of the obtained nanofibers after the electrospinning process. Before SEM measurements, the surface of the samples was coated (SC7620 sputter coater, Quorum Technologies Ltd., United Kingdom) with platinum for 120 s to obtain a conductive surface. The measurement of average fiber diameters was realized by using Image J software (National Institute of Health, USA). For this aim, 50 different points on SEM images were selected randomly for each fiber sample to reach the average fiber diameters, standard deviations, and distribution graphs of nanofiber diameters. High-resolution transmission electron microscopy (HR-TEM, Jeol 2100F) at 200 kV was used for further analysis of the morphology and crystalline structure of the calcined membrane. Prior to analysis, the calcined sample was prepared with a CF200-Cu Carbon film grid. First, the calcined sample was powdered with a pestle. The powder sample suspended in ethyl alcohol was mixed in an ultrasonic cleaner for 45 min, and then a drop was dropped on the grid with a micropipette and left to dry for one night. Electron dispersive spectroscopy (Thermo Scientific Axia ChemiSEM) at 8 kV was used to analyze the distribution of the elements in the calcined nanofiber membrane. The substitution of surface chemical species of the produced nanofiber membranes was investigated by X-ray photoelectron spectroscopy (XPS, Thermo Scientific K-Alpha) analysis. The Brunauer–Emmett–Teller (BET) surface area of each sample was analyzed by using liquid nitrogen with Micromeritics Gemini VII Version 2. Prior to analysis, samples were degassed at 110 °C for 8 h. N_2_ adsorption–desorption isotherms were measured in the relative pressure range (*P*/*P*_0_) 0.01–1. The Barrett–Joyner–Halenda (BJH) model by an adsorption isotherm was used to reach pore size distribution.

### 2.4 Antibacterial Activity

The antibacterial activity of the nanofiber membrane calcined at 750 °C was investigated against *Escherichia coli* BC1402 as a Gram-negative bacteria and *Staphylococcus aureus* ATCC 25923 as a Gram-positive bacteria using the quantitative viable count method modified by Akhtach et al.^56^ and Shi et al.^57^*E. coli* and *S. aureus* cells were inoculated into tryptic soy broth (TSB) and incubated for 18 h at 37 °C. Cells were harvested by centrifugation (5000 rpm, 10 min, 4 °C) and resuspended in an equal volume of physiological saline (0.85% NaCl), diluted to 1 × 10^6^ colony forming units (CFU)/mL after the incubation period.

All of the nanofiber membranes were sterilized in a UV cabinet for 30 min on each side. Nanofiber membranes (0.8 mg) were dispersed into the 2 mL of a sterile saline (0.85% NaCl) solution containing 10^6^ CFU/mL bacterial cultures. The suspension without a nanofiber membrane was used as the positive control. Each of the suspensions was incubated at 37 °C for 24 h on a rotary shaker at 300 rpm. After incubation, bacterial suspension was serially diluted in saline solution, plated on TSA, and incubated for 18–24 h at 37 °C. After incubation, the number of survival colonies was counted. All of the experiments were repeated twice with three parallels. The antimicrobial activities of the nanofiber membranes were calculated from the following equation.^54^
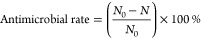
1where *N*_0_ and *N* indicate the average number of colonies in the control group and experimental group (CFU/mL), respectively.

### 2.5 Photocatalytic Degradation Studies

The photocatalytic activity of the obtained nanofiber membranes was investigated under both UV and simulated sunlight irradiation. Methylene blue (MB) was used as a model dye component for the photocatalytic activity measurements. Ceramic nanofibers (20 mg) were added to 40 mL of 20 mg/L aqueous methylene blue solution in the flask, and the solution was placed in a photoreactor on the mixer. For UV tests, the solution with a catalyst was exposed to UV light in a photoreactor which contains 18 UV-A (each lamp 8 W) lamps. On the other hand, 300 W Osram Ultra Vitalux with a cut-off filter was used as a simulated sunlight source and placed 20 cm away from the photoreactor. The temperature of the photoreactor was controlled with a fan. Prior to irradiation, the suspension of the catalyst in MB was stirred in the dark for 30 min to reach the adsorption–desorption balance. After that, 3 mL aliquots of the samples were taken every 10 min from the stirred solution, and the collected samples were filtered by a syringe filter. The MB absorbance values were measured at 664 nm with a UV–visible spectrophotometer (UV mini-1240 Shimadzu). The photocatalytic performance of the samples was evaluated by using the following equation.^21^
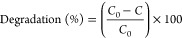
2where *C*_0_ is the initial MB concentration and *C* is the MB concentration after irradiation at a given time.

## 3 Results and Discussion

### 3.1 Structural Characterization

**Figure 1:**
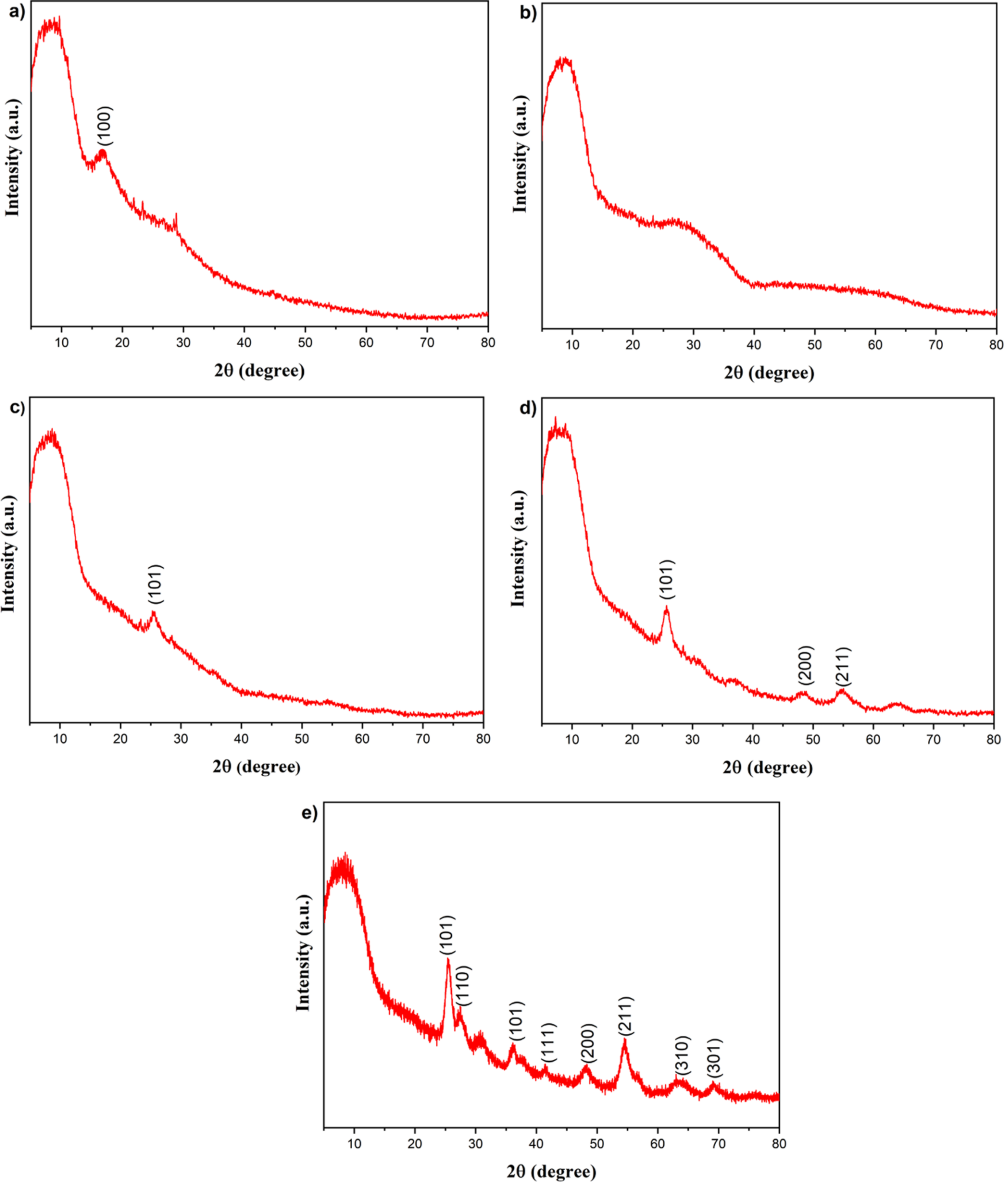
XRD patterns of the nanofibers (a) before calcination; after calcination at (b) 550 °C, (c) 650 °C, (d) 750 °C, and (e) 850 °C.

XRD analysis was conducted to assess the changes in the crystalline structure of the obtained nanofiber membranes. Figure 1 shows the XRD patterns of the membranes before and after the heat treatment processes. Before the calcination process (Figure 1a), the diffraction peak at 2θ of 16.5° corresponding to the crystal plane (100) represents the PAN linear macromolecules.^58^ When the membrane was calcined at 550 °C (Figure 1b), the disappearance of this diffraction peak indicates the removal of the PAN polymer with the heat treatment process. However, this temperature was relatively low for the formation of the anatase phase of TiO_2_. In the XRD pattern of the membrane, which was calcined at 650 °C, exhibited the reflection at 2θ of 25° corresponding to the (101) plane of the anatase phase (Figure 1c). When the calcination temperature was increased to 750 °C, additional peaks at the 2θ value of 48.2° corresponding to the (200) plane of the anatase phase and 2θ of 55.2° related to the (211) plane of the anatase phase (Figure 1d). Also, the number of peaks was highly increased with the increase of calcination temperature to 850 °C, and rutile phase formation started to occur (Figure 1e). The peaks were reached at 2θ values of 27.5, 36.2, 41, 54.6, 64, and 69.1° represented (110), (101), (111), (211), (310), and (301) planes of the rutile phase.^25,59−63^ Moreover, the peak at the 2θ value of 32° belongs to the brookite phase of TiO_2_.^64^ The lack of any other phase except TiO_2_ for the calcined membranes demonstrated that the dopants were well dispersed in the TiO_2_ lattice.

In this study, it is obviously seen that anatase to rutile phase transformation was delayed. Generally, pure TiO_2_ nanofibers have a tendency to form crystalline TiO_2_ fully at a calcination temperature of 450 °C. However, doping of TiO_2_ materials causes the increase of calcination temperature to the formation of crystalline TiO_2_.^30^ Researchers have studied different metal oxides and reached anatase to rutile phase transformation at higher temperatures.^14,30,65^ Li et al. studied hybrid SiO_2_ and TiO_2_ nanofiber calcined at 600 °C, and they did not reach the crystalline TiO_2_.^30^ In another study, Vieira et al. synthesized CeO_2_-doped TiO_2_ photocatalysts and reported that the increase in calcination temperature did not cause the change of the crystallinity of the material.^65^ The transformation of the anatase to rutile phase was inhibited in the Ce-doped TiO_2_ materials after heat treatment even at 600 °C. Kapusuz et al. reported that Zr doping to the TiO_2_ suppressed considerably the formation of the rutile phase.^14^

**Figure 2:**
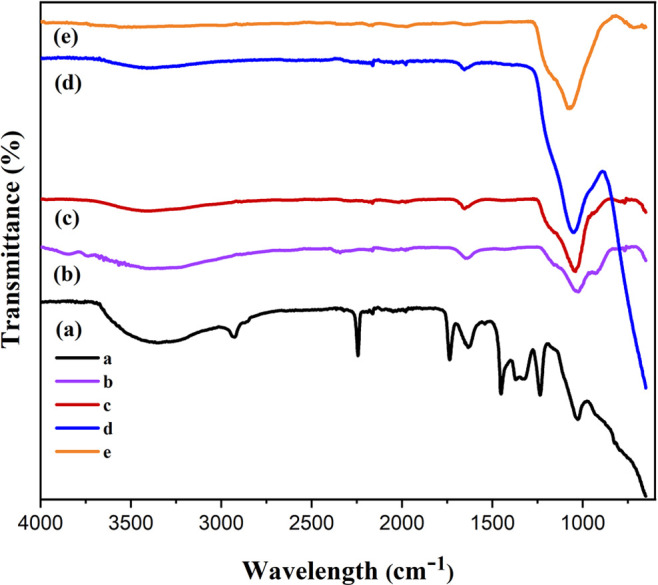
FT-IR results of the nanofibers (a) before calcination; after calcination at (b) 550 °C, (c) 650 °C, (d) 750 °C, and (e) 850 °C.

FT-IR analysis was performed to investigate the chemical composition of the obtained nanofiber membranes. Fourier transform infrared (FT-IR) graphs of the membranes, which were obtained before and after the calcination processes, are given in Figure 2. For all samples, the broad absorption band in the range of 3650–3100 cm^–1^ corresponded to H–OH stretching vibrations. The gradual decrease of this peak after the calcination process indicates the evaporation of hydroxyl groups.^66^ The absorption peak at 1630 cm^–1^ corresponded to hydrated species for the membrane before calcination, and this peak shifted to 1648–1654 cm^–1^ after the heat treatment process.^67^ When the FT-IR graph of the sample before the calcination process was examined (Figure 2a), the peak at 2925 cm^–1^ corresponds to the C–H stretching vibration of CH_2_ strains. The typical peak at 2244 cm^–1^ was assigned to the stretching vibrations of nitrile groups (−CN−) in PAN chains.^68^ Also, the peak at 1737 cm^–1^ belongs to the bending modes of adsorbed water. The peaks at 1452 and 1332 cm^–1^ indicated NO_3_^–1^ stretching. The peak at 1236 cm^–1^ corresponded to the −C=O stretching vibrations. All these peaks before the calcination process put forward the presence of the PAN polymer. The disappearance of all these peaks after the calcination process showed that the polymer was removed at a high temperature successfully (Figure 2b–e).^69^ On the other hand, the peak at 1027 cm^–1^ that was assigned to asymmetric ν(Si–O–Si) vibrations for the membrane before calcination shifted to the 1040–1073 cm^–1^ after the calcination process. However, the characteristic peak that belongs to the Si–O–Al vibration can be observed at the same wavelength, and thus, it was thought that this bond coincided with the asymmetric *ν*(Si–O–Si) and cannot be detected exactly.^67^

### 3.2 SEM–EDS Analysis

**Figure 3:**
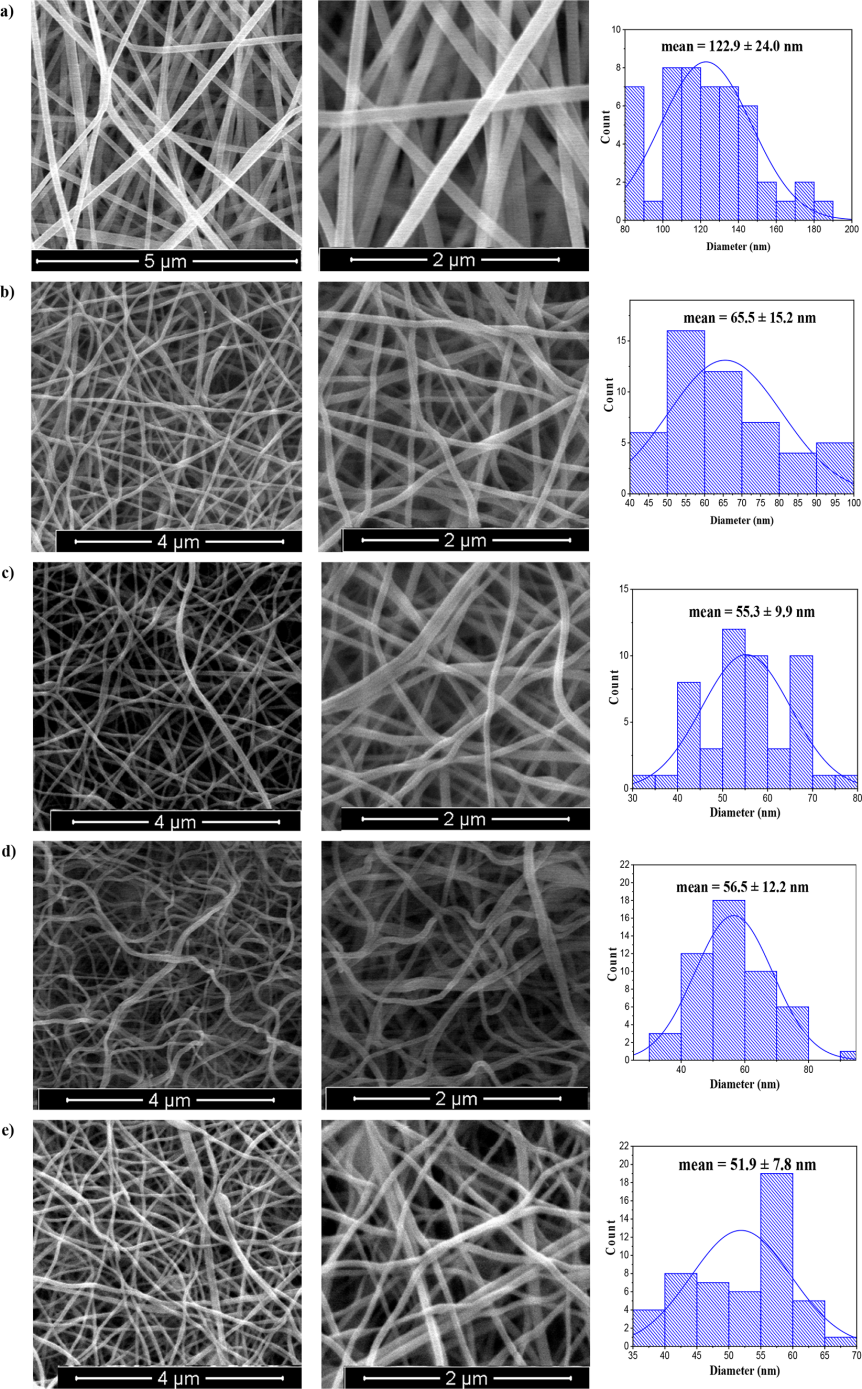
SEM images and histograms of diameters of the obtained nanofibers. (a) Before the calcination process, (b) 550 °C, (c) 650 °C, (d) 750 °C, and (e) 850 °C after calcination.

The surface morphologies of the as-fabricated nanofiber membranes were observed by SEM analysis. Figure 3 shows the SEM images with low and high magnifications and the distribution of diameters for each nanofiber membrane before and after the calcination processes. Before the calcination process, nanofibers distributed homogeneously without any bead structure, and the surface of the fibers was smooth with normal distribution (Figure 3a). The mean diameter was measured as 122.9 ± 24.0 nm. After heat treatment that was applied at different temperatures, the measured diameters of the membranes decreased and varied in the range of 51.9–65.5 nm owing to the destruction of the polymeric structure. Moreover, the obtained ceramic nanofiber membranes maintained integrity and homogenous distribution, although high calcination temperatures were applied to the nanofibers (Figure 3b–e). Since all electrospun ceramic nanofiber membranes had randomly oriented and uniformly distributed nanofibers with relatively low diameters, they can have high adsorption and photocatalytic degradation properties due to the high surface area to volume ratio.

**Figure 4:**
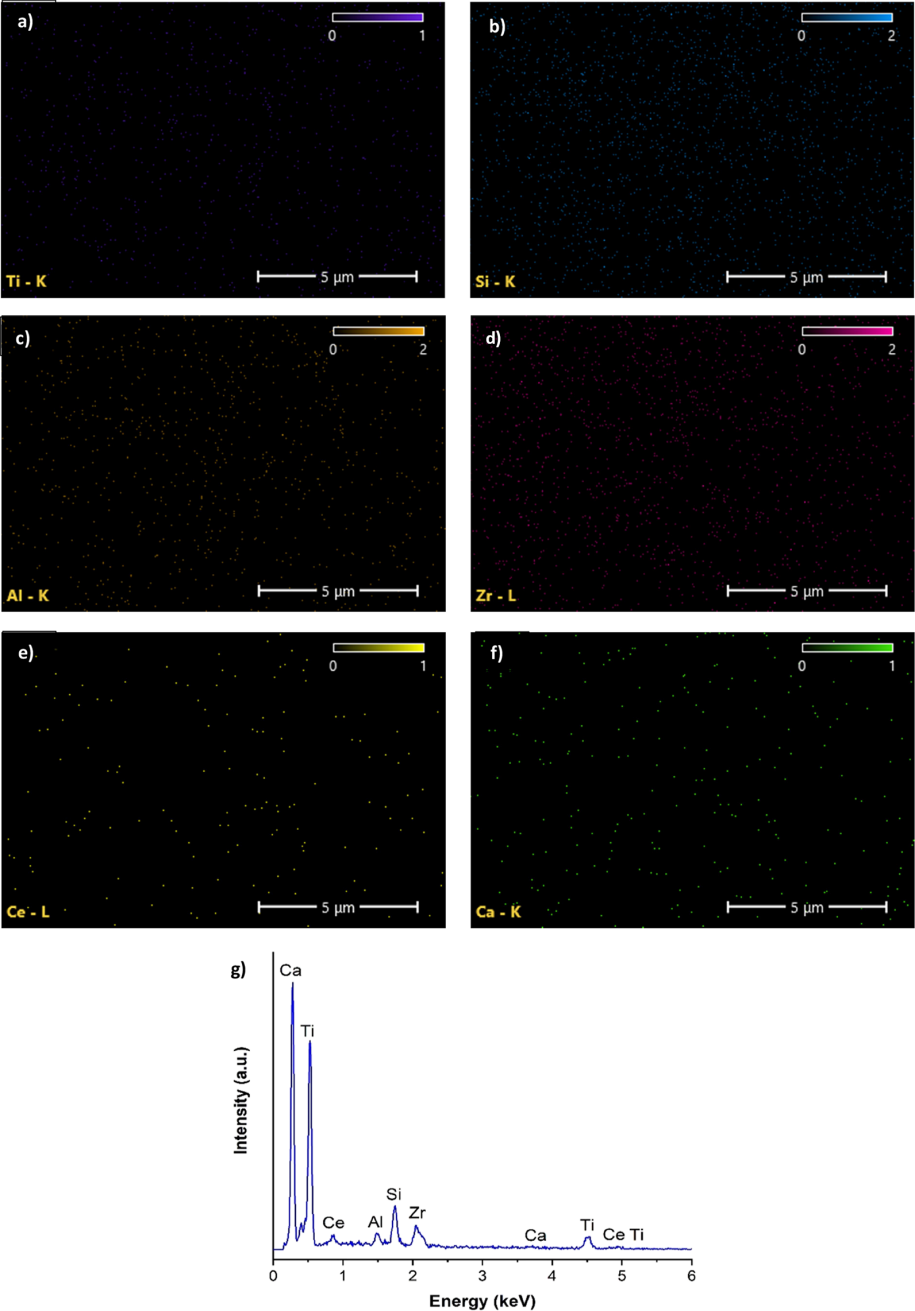
Elemental maps of (a) Ti, (b) Si, (c) Al, (d) Zr, (e) Ce, and (f) Ca. (g) EDS spectra of the nanofiber.

EDS mapping results and spectra can be seen from Figure 4. All elements were uniformly distributed throughout the whole ceramic nanofiber. The well-mixed heterojunction, which was composed of Ti, Si, Zr, Al, Ca, and Ce, was verified (Figure 4a–f). The co-existence of these elements was further confirmed with the EDS spectrum taken as a representative area from nanofibers (Figure 4g). The resulting elemental ratios were found to be similar to theoretical synthesis ratios, and it was demonstrated that the structure mainly contains high TiO_2_, which is in accordance with XPS analysis.

### 3.3 HR-TEM Analysis

**Figure 5:**
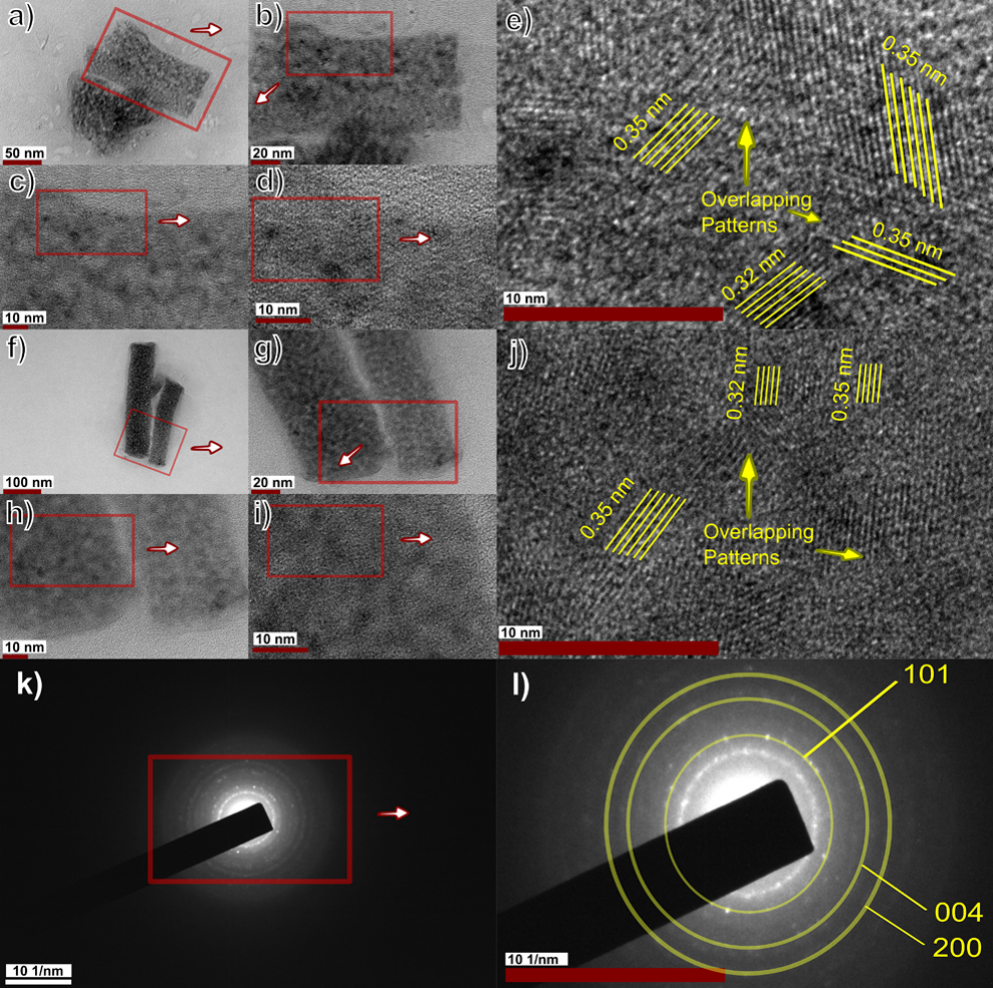
HR-TEM and SAED images of the calcined nanofiber at 750 °C. (a–e: HR-TEM images of the nanofiber junction with increasing magnification. f–j: HR-TEM images of the nanofiber body with increasing magnification. k, l: SAED images with increasing magnification and Miller index values.)

The HR-TEM images taken from different sections of the produced sample are given in Figure 5a–j with highlighted parameters. The different sets of clear lattice fringe formations with various directions mainly formed with *a d*-spacing of 0.35 nm corresponding to the anatase phase of the TiO_2_ (101) crystal plane. Also, the formation of dark zones (Figure 5d) with *a d*-spacing of 0.32 nm related to the rutile phase of the TiO_2_ (110) crystal plane.^70,71^ This result confirmed the only presence of the TiO_2_ crystal lattice fringe in the ceramic nanofiber structure. As a result of this, ingredients other than TiO_2_ were embedded in anatase crystal planes with little disturbance to the orientation. The difference between gray and white zones showed the existence of overlapping direction patterns. The highlighted locations in Figure 5e,j indicated the overlapping of more than two crystal patterns. Therefore, the thickness of nanofiber walls increased in similar locations.

The selected area electron diffraction (SAED) pattern of the sample (Figure 5k,l) indicated the high crystallinity of anatase TiO_2_ in the obtained ceramic nanofiber structure. The ring-type diffraction pattern implied the well-crystallized structure of the nanofiber without the formation of any other phases.^71,72^ The radius of apparent circles was measured and matched with Miller indices and diffraction angles (2θ). According to the measurements (Table 1), SAED results were similar to XRD results in Figure 1d showing the anatase phase of TiO_2_.^73^

**Table 1:** SAED Circle Radius, Diffraction Angles, and Equivalent Miller Indices

circle radius (nm)	diffraction angle (2θ)	Miller indices (*h*-*k*-*l*)
5.8	25.7	1-0-1
8.6	38.1	0-0-4
10.5	46.5	2-0-0
12.7	55.9	2-1-1

### 3.4 BET Surface Area

Specific surface area investigations of the obtained samples were performed by BET analysis. Table 2 shows the BET surface area, Barrett–Joyner–Halenda (BJH) pore size, and pore volume results of the membranes that calcined at different temperatures. Since the shrinkage of the ceramic matrix induced the enlargement of pores, the specific surface area decreased with increasing temperature. This result is coincident with the other related studies on TiO_2_-based materials for photocatalysis.^43,74^ The high surface area to volume ratio induces to increase in adsorption capacity, and thus, more organic pollutants could be adsorbed on TiO_2_-based nanofiber materials. BET surface areas of the obtained nanofibers (82.9–149.2 m^2^/g) except the calcined membrane at 850 °C were found to be higher than that of commercial TiO_2_ (50 m^2^/g), which indicated the improvement of adsorption and photocatalytic properties.^75^

**Table 2:** Textural Properties of the Membranes at Different Heat Treatment Temperatures

calcination temperature (°C)	BET surface area (m^2^/g)	BJH pore size (nm)	BJH pore volume (cm^3^/g)
550	149.2	7.036	0.209
650	111.0	9.579	0.245
750	82.9	9.908	0.197
850	46.6	12.692	0.142

**Figure 6:**
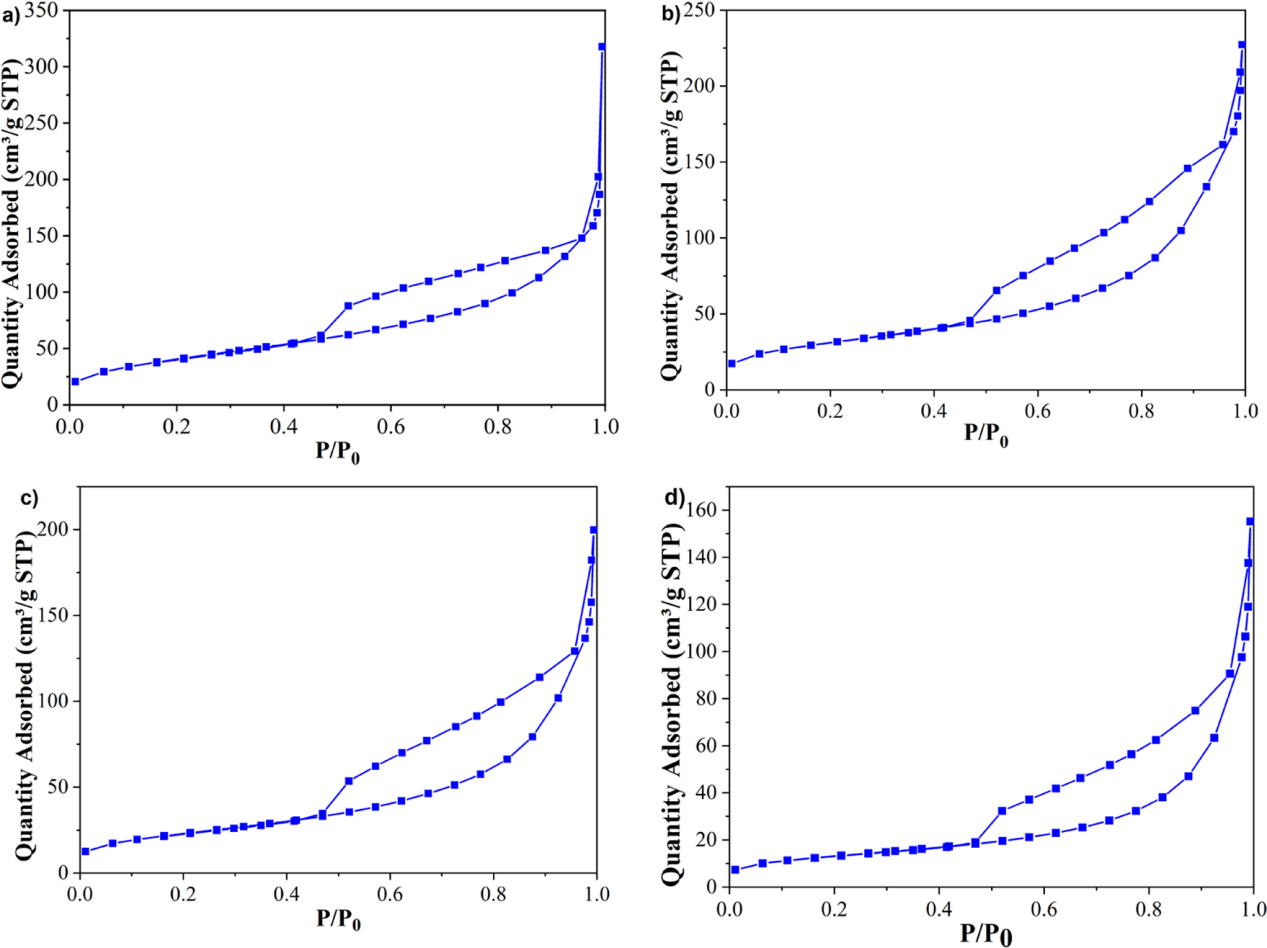
N_2_ adsorption–desorption isotherms of membranes calcined at (a) 550 °C, (b) 650 °C, (c) 750 °C, and (d) 850 °C.

Nitrogen adsorption–desorption isotherms of the calcined membranes can be seen from Figure 6. According to the IUPAC classification, both calcined membranes present a direct hysteresis loop that belongs to the type IV isotherm. These results indicated the mesoporous structures of the obtained samples.^74,76^

### 3.5 Photocatalytic Activity

**Figure 7:**
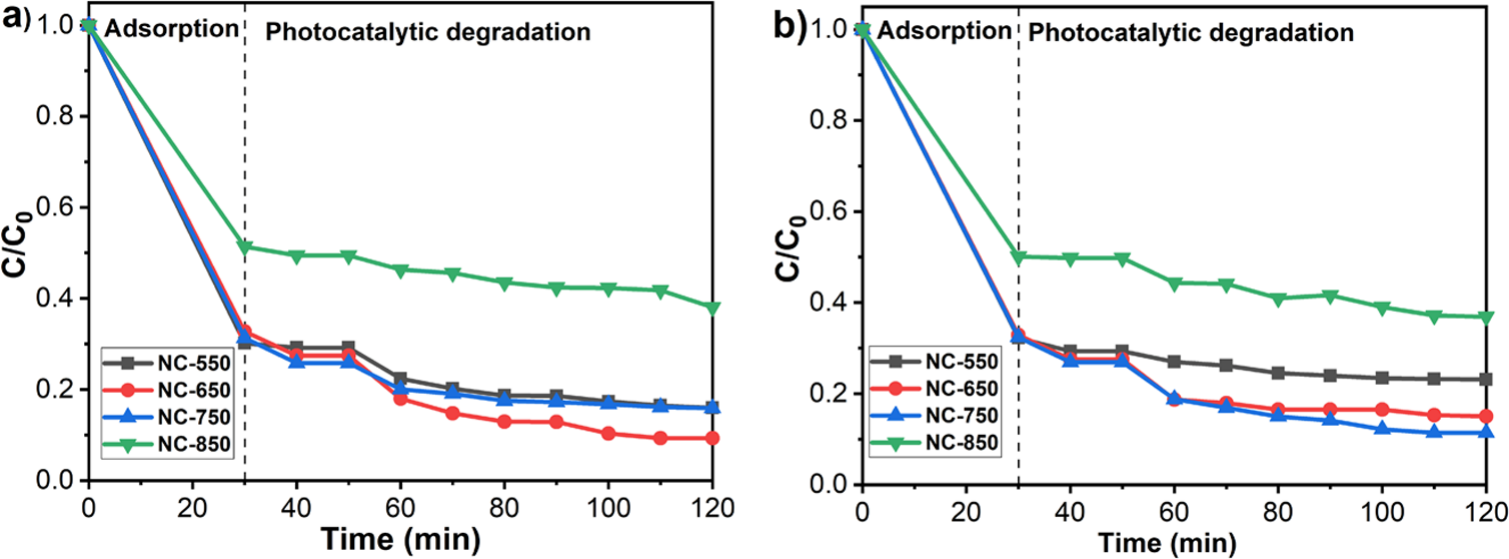
Time-dependent degradation efficiency of nanofiber membranes under (a) UV, and (b) simulated sunlight irradiation.

Methylene blue (MB) is a cationic dye that is used in different industries such as coloring, textile, and petrochemical industries, which leads to water pollution. The removal of this pollutant dye from wastewater is a big issue for human health and the environment.^77^ In this study, the photocatalytic degradation of 20 ppm MB with prepared nanofiber membranes was investigated under both UV and simulated sunlight irradiation. First, photocatalysts were stirred in the dark for 30 min to reach adsorption–desorption equilibria. The concentrations of MB decreased remarkably for all samples, as can be seen in Figure 7, due to the high adsorption capacity of the obtained nanofiber membranes. According to calcination temperature, the calcined nanofibers were coded as NC-550, NC-650, NC-750, and NC-850. The adsorption step is the essential process for heterogeneous photocatalysis that stimulates the interaction between dye molecules and photocatalysts, and thus, the degradation of the adsorbed dye molecules on the photocatalyst surface could be realized more easily.^78,79^ The high adsorption capacity of the fabricated nanofiber membranes indicated that a large amount of MB dye molecules were exhibited on the surface. Therefore, the superoxide anion radicals and hydroxyl radicals, which were generated from electron (e−)-hole (h+) coupled reaction with oxygen and water, ensured the possibility of reaching a higher degradation ratio for MB molecules.^21,61^ After the adsorption–desorption process was completed, the photocatalytic performances of the nanofiber membranes were investigated by opening UV and sunlight resources for 90 min of exposure time on the samples. As can be seen in Figure 7, the maximum degradation efficiency was achieved with the ceramic nanofiber material calcined at 650 °C under UV light (90.7%), while it was achieved with the sample calcined at 750 °C under simulated sunlight irradiation (88.5%). Although adsorption capacity was found as very high at the calcination temperature of 550 °C, the photocatalytic degradation efficiency was lower than that of other calcined samples at 650 and 750 °C. The reason for this lower efficiency was that there was no anatase phase formation at this temperature, which was confirmed with XRD analysis. The surface area of nanomaterials plays an important role in adsorption capacity and photocatalytic degradation efficiency. Generally, the high surface area provides to increase the photocatalytic efficiency of nanomaterials by ensuring the adsorption of more pollutants onto the nanomaterial surface. On the other hand, photocatalytic activity depends on not only the surface area but also the structural, morphological, and optical properties of nanomaterials. Since the amorphous TiO_2_ enables electron–hole recombination on the surface easily, TiO_2_ in the anatase phase is considered to show higher photocatalytic activity than TiO_2_ in an amorphous structure.^61^ On the other hand, the nanofiber membrane calcined at 850 °C has the lowest adsorption capacity and photocatalytic degradation efficiency compared to other calcined membranes due to having the lowest surface area and the rutile phase structure. These results revealed that both the crystalline structure and surface area played a significant role in determining the photocatalytic efficiency of the fabricated nanofiber materials.

**Figure 8:**
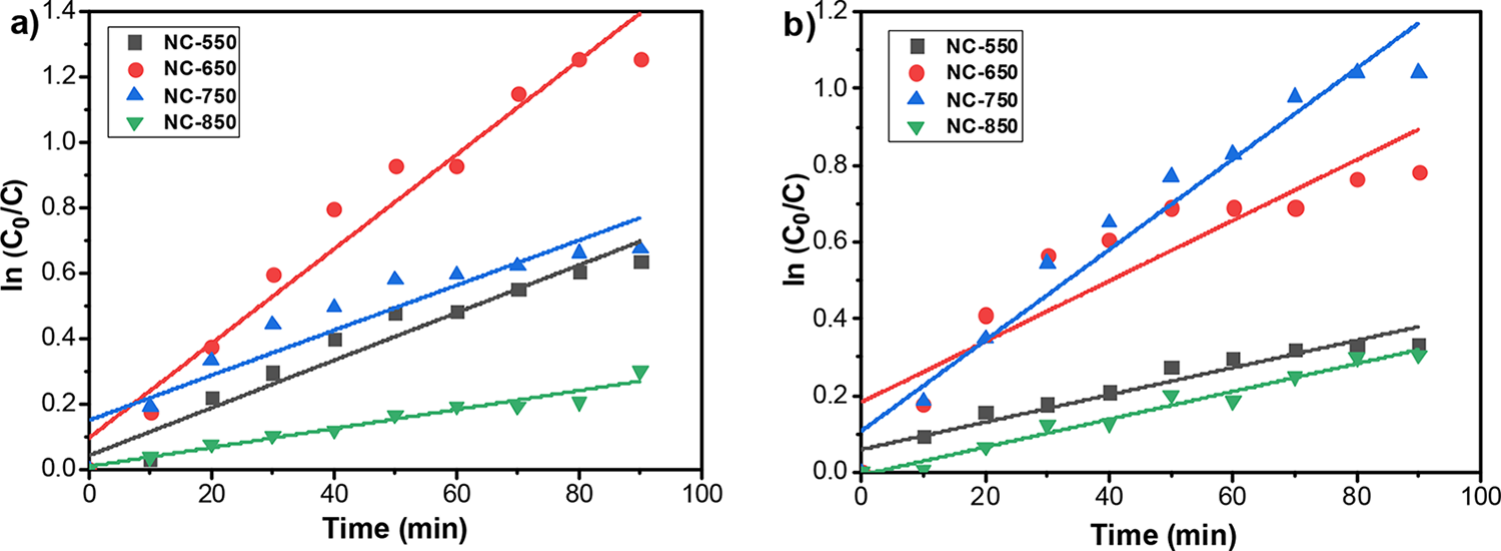
Kinetic data of the nanofiber membranes for the photocatalytic degradation of MB under (a) UV, and (b) simulated sunlight irradiation.

The photodegradation rate constants of MB by utilizing the prepared samples calcined at different temperatures conformed with the pseudo-first order kinetics:^77^
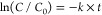
3where *C* and *C*_0_ are the concentration of the sample after a given time *t* and 0, respectively, *t* is light irradiation time, and *k* is the reaction rate constant. The photodegradation behavior of the samples was identified with the Langmuir–Hinshelwood mechanism, which could be obtained from the relationship of ln (*C*/*C*_0_) versus irradiation time (*t*) for MB (Figure 8). Rate constants were reached from the slope of linear fitting the data, as shown in Figure 8, for each sample calcined at different temperatures and are listed in Table 3. The highest removal rate for MB was accomplished with the nanomaterial calcined at 650 °C under UV light, while it was reached with the sample calcined at 750 °C under sunlight irradiation. The reason for this was discussed above, and the results were found to be coherent with the photodegradation efficiencies. The rate of photocatalytic reactions is influenced by various factors, such as calcination temperature. The increasing calcination temperature may lead the conflicting impacts on the surface area and crystallinity of the photocatalysts, which result in an unpredictable effect on photocatalytic degradation rates.^80^ In this study, the rate of reaction was accelerated with increasing calcination temperature up to a range of anatase to rutile phase transformation of TiO_2_, which is one of the most important factors in reaching the high degradation rates for containing TiO_2_ materials. This result was also confirmed by the faster lightening of the blue color of MB during the experiment. The clear improvement of the photocatalytic degradation efficiency for MB removal was achieved by using the synergistic effect of different dopants on the TiO_2_ nanofibers. The photocatalytic properties of bare TiO_2_ and doped TiO_2_ materials have been reported in different studies. The degradation efficiencies and the related rate constants differ from each other owing to the changing operational conditions. Sanguino et al. studied MB photocatalytic degradation under visible light, and they resulted in the photocatalytic degradation rate constant as 100 times higher than the bare TiO_2_ (5 × 10^–5^ min^–1^).^81^ Moongraksathum et al. synthesized TiO_2_–CeO_2_ films to investigate the MB degradation and measured the first-order rate constants as 0.552 and 0.283 h^–1^ for CeO_2_-doped TiO_2_ films under UV and visible light irradiation, respectively, while they reached the half of the rate constants for undoped TiO_2_ film.^82^ Abdi et al. added ZrO_2_ to the TiO_2_ structure for the photodegradation of Rhodamine B, and they reached the first-order rate constants as 0.0036 min^–1^ for pure TiO_2_ and 0.013 min^–1^ for the TiO_2_/ZrO_2_ photocatalyst at 180 min.^83^ Hussain et al. investigated the MB dye degradation performance of the metal organic framework-derived Co_3_O_4_@ZnO nanomaterial, and they reached 55% MB degradation at 80 min reaction time under visible light irradiation.^84^ When compared with the literature studies, it is obviously seen that promising results were obtained with the doped nanofibers containing anatase TiO_2_ calcined at 650 and 750 °C under both UV and sunlight irradiation.

**Table 3:** Pseudo-First Order Rate Constants and Regression Coefficients of the Nanofiber Membranes Depend on the Calcination Temperature

sample code	*k* (min^–1^) - UV	*R*^2^	*k* (min^–1^) - sunlight	*R*^2^
NC-550	0.00728	0.946	0.00355	0.920
NC-650	0.01444	0.962	0.00790	0.826
NC-750	0.00688	0.874	0.01181	0.959
NC-850	0.00290	0.960	0.00363	0.974

### 3.6 XPS Analysis

**Figure 9:**
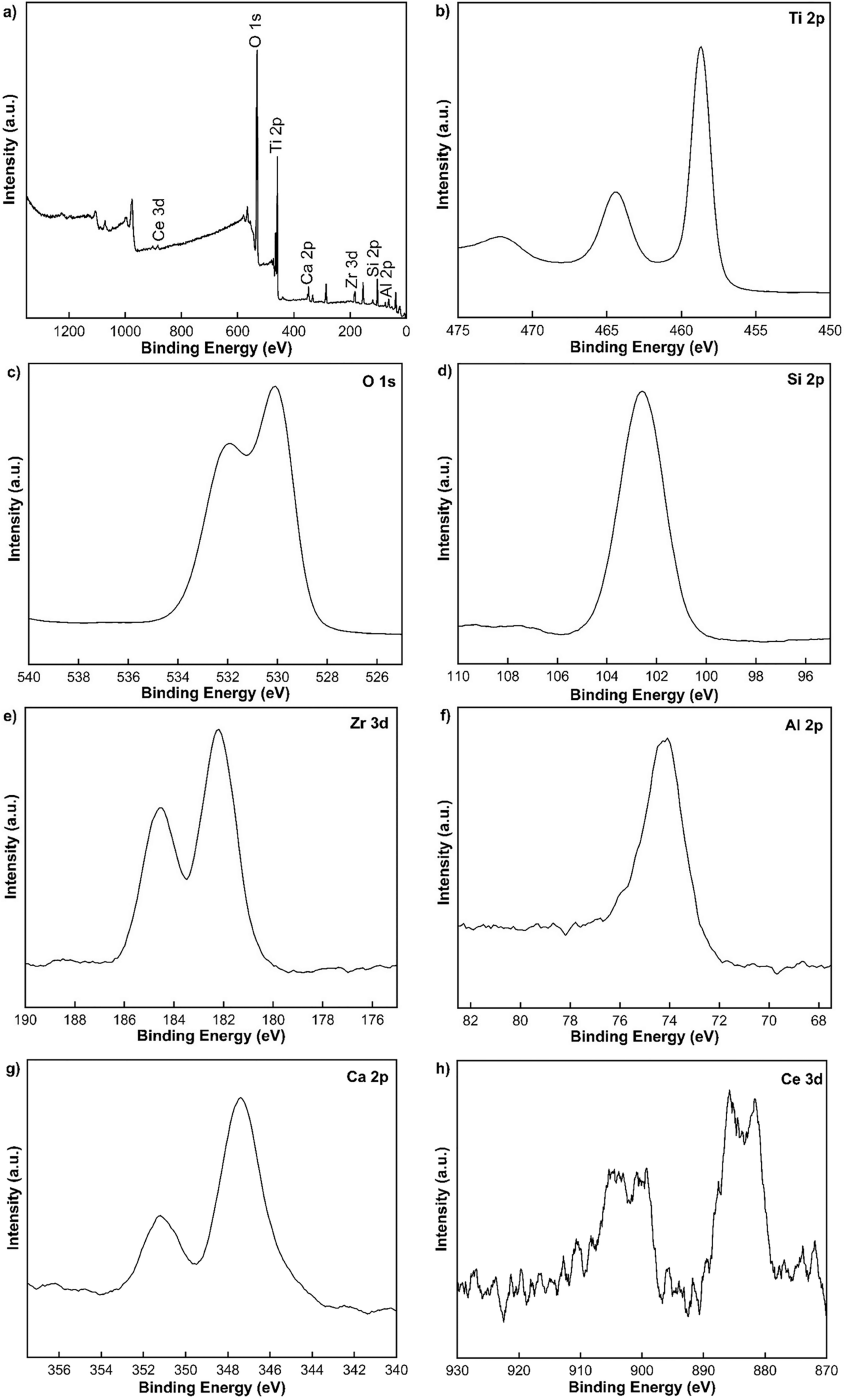
XPS spectra of the nanofiber membrane (a) full survey scan, (b) Ti 2p, c) O 1s, (d) Si 2p, (e) Zr 3d, (f) Al 2p, (g) Ca 2p, and (h) Ce 3d.

The XPS analysis was performed to obtain the surface chemical species of the as-prepared nanofiber membrane that calcined at 750 °C. Since the maximum photocatalytic efficiency for the degradation of 20 ppm methylene blue under simulated sunlight irradiation and the excellent nanofiber morphology (bead-free structure) was reached by using the membrane calcined at 750 °C, XPS analysis was applied by using this membrane. As seen in Figure 9a, the presence of Ti, O, Si, Zr, Al, Ca, and Ce in the fabricated sample was confirmed with a full survey scan. The high-resolution XPS spectra of the Ti 2p, O 1s, Si 2p, Zr 3d, Al 2p, Ca 2p, and Ce 3d are given in Figure 9b–h. For the XPS spectra of Ti 2p, the two diffraction peaks exhibited at 464.48 and 458.68 eV, relating to Ti 2p1/2 and 2p3/2, and this result demonstrated the presence of Ti^4+^ (Figure 9b).^85^ Since no peak of Ti^3+^ was observed, it was concluded that TiO_2_ was quite stable in the system.^54^Figure 9c displays the O 1s spectra with the peaks at 530.08 and 531.18 eV, which corresponded to the lattice oxygens and surface hydroxyl groups.^86^ In Figure 9d, the single observed peak at 103.58 eV can be attributed to the Si 2p.^76,79^ As shown in Figure 9e, the peaks exhibited at 182.18 and 184.58 eV can be assigned to the Zr 3d5/2 and Zr 3d3/2, respectively.^80^Figure 9f shows the Al 2p spectra, which is related to the peak at 74.87 eV.^87^ As displayed in Figure 9g, the two spin-orbital components exhibited at 347.38 and 351.18 eV corresponded to the Ca 2p spectrum.^53^ In Figure 9h, the two diffraction peaks at 885.78 and 900.98 eV can be attributed to the Ce 3d5/2 and 3d3/2 with the Ce^3+^ oxidation state, respectively. On the other hand, the observed peaks at 899.38 and 916.88 eV related to CeO_2_, which indicated the presence of the Ce^4+^ oxidation state.^87,88^ The coexistence of Ce^3+^ and Ce^4+^ oxidation states is beneficial for the surface reactions by supporting reactive oxygen species (ROS) formation in photocatalytic processes.^54,87^

### 3.7 Antibacterial Activity

**Figure 10:**
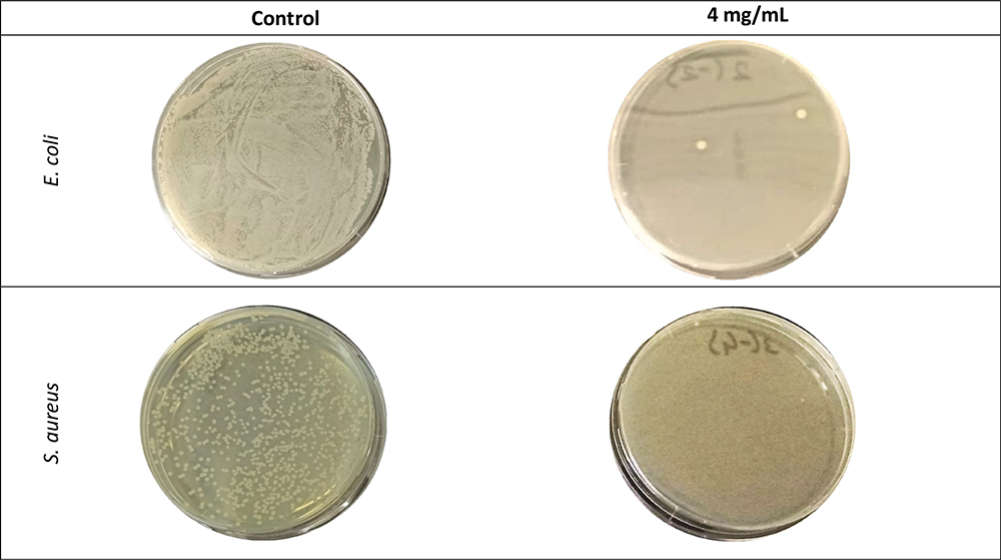
Petri images of *E. coli* and *S. aureus* after 24 h of incubation from the control and nanofiber samples at the same dilution.

The antibacterial ability of the nanofiber membrane calcined at 750 °C was tested against two different bacteria: *Escherichia coli* BC 1402 as a Gram-negative bacterium and *Staphylococcus aureus* ATCC 25923 as a Gram-positive bacterium. The viable cell count method was used, and the concentration of the nanofibrous membrane in microbial culture was arranged as 4 mg/mL. The number of viable bacteria in the presence of nanofiber membranes was found as 8.01 × 10^3^ CFU/mL for *E. coli* and 1.42 × 10^4^ CFU/mL for *S. aureus*, while the positive control sample had 1.3 × 10^7^ CFU/mL for *E. coli* and 1.59 × 10^7^ CFU/mL for *S. aureus*. The photographs of agar plates spread with the control cell suspension and those subjected to nanofiber membrane are given in Figure 10. The obtained nanofibrous membranes exhibited a very high antimicrobial rate of 99.94 and 99.91% against *E. coli* and *S. aureus*, respectively. Also, the results of these bacterial inhibitions are stated as the logarithmic decrease (log reduction), as shown in Table 4. However, the obtained nanofibrous membrane exhibited highly promising inhibition of both *E. coli* and *S. aureus*. Gram-positive (*S. aureus*) microorganisms were found to be more resistant to the nanofiber membrane. The reason for the higher inhibition rate of Gram-negative (*E. coli*) microorganisms when compared with Gram-positive (*S. aureus*) microorganisms was the thicker peptidoglycan layer of *S. aureus* which coincided with literature studies. The difference between the cell wall structures of the two microorganisms changed the diffusion properties.^89,90^

**Table 4:** Antibacterial Test Results of Nanofiber Membranes on Gram-Negative (*E. coli*) and Gram-Positive (*S. aureus*) Microorganisms

	*E. coli*		*S. aureus*	
	control	4 mg/mL sample	control	4 mg/mL sample
log (CFU/mL)	7.11	2.90	7.20	4.15
log reduction		4.22		3.06

In literature studies, the antibacterial activities of TiO_2_ and doped TiO_2_ materials were investigated under UV and sunlight irradiation in general.^86,91,92^ In a study, TiO_2_ nanomaterials did not show visible inhibitory effect against tested microorganisms; this result was explained with low diffusion and solubility in the solid medium.^93^ However, Zr-doped TiO_2_ nanoparticles showed higher antibacterial activity by the formation of reactive oxygen species (ROS).^52^ The antibacterial mechanism of metal oxide nanofiber membranes occurs through the formation of ROS under both UV and dark conditions. However, as can be seen on nanofiber materials containing TiO_2_, there is a positive relationship between light irradiation and antibacterial activity due to the enhancement of ROS production.^94^ In addition, metal doping decreases the crystalline size, which enables higher antibacterial activity.^52^ In contrast, the main reason for the high inhibition rate of microorganisms by the obtained nanofiber membrane was the reaction with the cell wall, which led to the killing of bacteria. In this study, the synergistic effect of doping materials provided to reach efficient antibacterial activity despite the absence of light irradiation.^86,91^

## 4 Conclusions

Highly efficient TiO_2_-based multi-oxide nanofiber membranes were successfully fabricated via sol–gel and electrospinning methods in the system of TiO_2_–SiO_2_–Al_2_O_3_–ZrO_2_–CaO–CeO_2_ and calcined at different temperatures from 550 to 850 °C. XRD analysis revealed that the low calcination temperature of 550 °C was not enough for anatase phase formation for TiO_2_, and anatase to rutile phase transformation was observed at a high temperature of 850 °C. Moreover, the anatase cyrstalline formation was determined by HR-TEM. Due to the synergistic effect of doping materials to TiO_2_, remarkably high total degradation rates of MB were achieved. The photocatalyst calcined at 650 °C showed the highest MB degradation rate under UV, whereas a similar result was obtained with the membrane calcined at 750 °C under sunlight irradiation. Although the specific surface area decreased with increasing calcination temperature, the photodegradation rate of MB was enhanced up to a certain level of crystallinity of nanofiber membranes. It could be inferred that both the degree of crystallinity and surface area impressed the adsorption capability and photocatalytic performance of nanofiber membranes. In addition to these superior properties, the membrane calcined at 750 °C exhibited very high inhibition rates against *E. coli* and *S. aureus*. The synergistic effect of doping materials not only improved photodegradation properties but also ensured the inhibition of the growth of microorganisms such as *E. coli* and *S. aureus*. Furthermore, compared with TiO_2_-coated photocatalytic materials, the direct incorporation of TiO_2_ in glass ceramic systems prevented the peeling problem. To sum up, an excellent 3D multi-oxide doped TiO_2_ ceramic nanofiber, which can be used in various applications requiring both antibacterial and photocatalytic properties, was obtained in this study. Future studies will be conducted to the obtained sample in a continuous process to be able to see the long-term stability and the efficiency of the sample for the waste-water treatment systems.
